# Evaluation of the dual-action tissue closure device for perforation closure after endoscopic full-thickness resection of gastric submucosal tumors >10 mm

**DOI:** 10.1093/gastro/goag070

**Published:** 2026-07-21

**Authors:** Lingjun Meng, Jianqun Cai, Zhen Wang, Baohua Zhang, Zhiru Gao, Jihua Chen, Dongbo Zhang, Panpan Liu, Tongyin Xing, Yan Xu, Shanci Wang, Yan Meng, Side Liu, Qiang Zhang

**Affiliations:** Department of Gastroenterology, Nanfang Hospital, Southern Medical University, 1838 Guangzhou Avenue North, Baiyun District, Guangzhou, Guangdong, China; Department of Gastroenterology, Nanfang Hospital, Southern Medical University, 1838 Guangzhou Avenue North, Baiyun District, Guangzhou, Guangdong, China; Department of Gastroenterology, Nanfang Hospital, Southern Medical University, 1838 Guangzhou Avenue North, Baiyun District, Guangzhou, Guangdong, China; Department of Gastroenterology, Nanfang Hospital, Southern Medical University, 1838 Guangzhou Avenue North, Baiyun District, Guangzhou, Guangdong, China; Department of Gastroenterology, Nanfang Hospital, Southern Medical University, 1838 Guangzhou Avenue North, Baiyun District, Guangzhou, Guangdong, China; Department of Gastroenterology, Nanfang Hospital, Southern Medical University, 1838 Guangzhou Avenue North, Baiyun District, Guangzhou, Guangdong, China; Department of Gastroenterology, Nanfang Hospital, Southern Medical University, 1838 Guangzhou Avenue North, Baiyun District, Guangzhou, Guangdong, China; Department of Gastroenterology, Nanfang Hospital, Southern Medical University, 1838 Guangzhou Avenue North, Baiyun District, Guangzhou, Guangdong, China; Department of Gastroenterology, Nanfang Hospital, Southern Medical University, 1838 Guangzhou Avenue North, Baiyun District, Guangzhou, Guangdong, China; Department of Gastroenterology, The First Affiliated Hospital of Guangzhou Medical University, Guangzhou Medical University, 151 Yanjiang West Road, Yuexiu District, Guangzhou, Guangdong, China; Department of Gastroenterology, Nanfang Hospital, Southern Medical University, 1838 Guangzhou Avenue North, Baiyun District, Guangzhou, Guangdong, China; Department of Gastroenterology, Nanfang Hospital, Southern Medical University, 1838 Guangzhou Avenue North, Baiyun District, Guangzhou, Guangdong, China; Department of Gastroenterology, Nanfang Hospital, Southern Medical University, 1838 Guangzhou Avenue North, Baiyun District, Guangzhou, Guangdong, China; Department of Gastroenterology, Nanfang Hospital, Southern Medical University, 1838 Guangzhou Avenue North, Baiyun District, Guangzhou, Guangdong, China; Department of Gastroenterology, Zengcheng Branch, Nanfang Hospital, Southern Medical University, Guangzhou, Guangdong, China

**Keywords:** endoscopic full-thickness resection (EFTR), dual action tissue closure device (DAT), perforation closure, gastric submucosal tumors (G-SMTs)

## Abstract

**Background:**

Endoscopic full-thickness resection (EFTR) is an effective technique for endoscopic resection of gastric submucosal tumors (G-SMTs); however, the closure of the post-operative perforation wound presents a significant challenge. We invented a dual action tissue closure device (DAT), and aimed to evaluate its feasibility and safety in closing perforation wounds following EFTR of G-SMTs.

**Methods:**

This is a retrospective study that included the data of patients who underwent EFTR for G-SMTs at Nanfang Hospital (Guangzhou, China) between 1 January 2021 and 1 April 2024. All these wounds were closed using the DAT and through-the-scope clip. The collected data included tumor size and location, total procedure time, closure success rate, closure time, complications, and follow-up outcomes. The closure efficacy of the DAT was evaluated accordingly.

**Results:**

Twenty-nine patients (median age, 57 years old, 37.9% women) completed EFTR of G-SMTs, which included 2 cases utilizing cap-assisted full-thickness resection and 27 cases using EFTR. The tumors had a median long diameter of 20 (15–30) mm and short diameter of 15 (10–25) mm. All wounds were successfully closed without complications. The median total operation time was 55 minutes (interquartile range: 33.5–70.0 minutes), and the median wound closure time was 19 minutes (range: 12.0–27.0 minutes). The median number of DAT used was 1 (interquartile range: 1–3), with a median operation time of 3 minutes (1.6–6.5) for per DAT. Seven patients underwent endoscopic follow-up, with 88.2% of the DATs falling off naturally.

**Conclusions:**

DAT can effectively and safely close perforation wounds following EFTR of G-SMTs.

## Introduction

Endoscopic full-thickness resection (EFTR) has emerged as a viable treatment option for gastric submucosal tumors (G-SMTs), including gastrointestinal stromal tumors, leiomyomas, and lipomas [1]. Currently, G-SMTs with a diameter of < 30 mm are considered suitable for EFTR [2]. It is crucial to ensure an adequate margin of resection to achieve microscopically negative edge resection (R0) [3]. Complete tumor resection minimizes the risk of residual tumor and recurrence [4]. EFTR for SMTs achieves a 100% complete resection rate with extremely low complication rates [5].

However, the rapid and reliable closure of perforation wounds after EFTR remains a challenge [6]. Various factors contribute to this difficulty, including the thick and slippery nature of the gastric mucosa, high mucosal tension, and the compression of the gastric cavity post-perforation. Currently, the traditional through-the-scope clip (TTSC) is more suitable for closing gastric wounds <10 mm in diameter, while over-the-scope clips can successfully close gastric wounds with a diameter of 10–20 mm [7, 8]. Additionally, endoscopic purse-string suturing [9] and other advanced endoscopic techniques for large wound closure cannot be directly implemented with conventional endoscopic instruments, which limits the efficiency of suturing. For perforation wounds after EFTR, efficient and firm closure directly impacts the patient safety, making instruments that can pass through the endoscopic working channel without requiring scope withdrawal the preferred choice.

**Figure 1:**
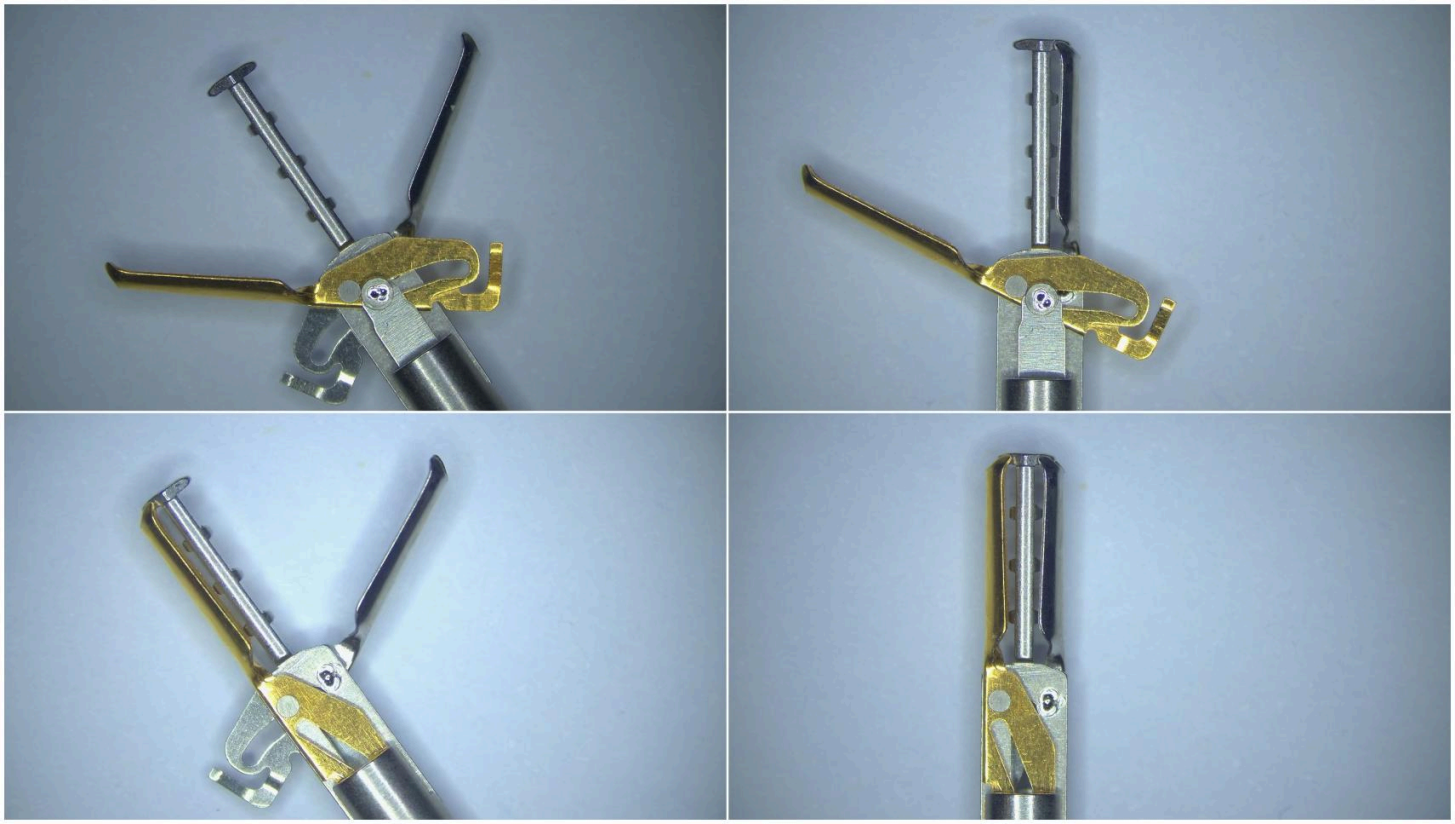
Physical image of DAT. By using our hands to operate the handle of DAT, the metal clips on both sides can be opened and closed independently. DAT, dual action tissue closure device.

Dual action tissue closure device (DAT) was developed to effectively close large gastrointestinal wounds (Figure 1) [10, 11]. It can be introduced through the endoscopic working channel without requiring scope withdrawal for installation. In 2021, we reported the first successful case of closure of a perforation wound (diameter 2.5 cm) in the gastric fundus using DAT and TTSC [12]. The total closure time was 15 minutes, with an average of 2.5 minutes per DAT application, and no complications were observed. This indicates that the combination of DAT and TTSC is a promising approach for closing perforation wounds following EFTR [13]. As we have gained experience with DAT, we have compiled cases of gastrointestinal tract lesions (G-SMTs) larger than 1 cm, which were sutured using DAT and TTSC, to preliminarily assess its effectiveness and safety.

## Patients, material, and methods

This retrospective study analyzed continuous cases of G-SMTs treated with EFTR and gastric perforation closure using DAT at the Digestive Endoscopy Center of Southern Hospital between 1 January 2021 and 1 April 2024. The device used, DAT, received approval from the National Medical Products Administration of China and is currently marketed. Furthermore, this study has been ethically reviewed and approved by the Ethics Committee of Southern Medical University (No. NFEC-2025–128).

All procedures were conducted by four experienced endoscopic physicians, each of whom has completed between 50 and over 1,000 endoscopic submucosal dissection (ESD) surgeries. At the study’s inception, one of the endoscopists was the developer of DAT and possessed experience in its application, while the other physicians had no prior experience using DAT for wound closure.

If perforation occurred during ESD, abdominal paracentesis was performed based on intra-abdominal pressure. Following disinfection and local anesthesia at the contralateral McBurney’s point, a 16‑gauge needle connected to a sterile syringe was inserted vertically under gentle negative pressure. Peritoneal entry was confirmed by loss of resistance and free aspiration, after which decompression was performed. A gastric decompression tube was inserted postoperatively at the surgeon’s discretion. After recovery from anesthesia, analgesia with intramuscular tramadol 0.1 g was administered as clinically indicated.

### Endoscopic resection procedure and equipment

Except for the two patients who underwent cap-assisted full-thickness resection (Cap-EFTR) [14], all other patients completed the surgery under general anesthesia with endotracheal intubation. Carbon dioxide was injected during all surgeries. Prior to the procedures, all cases underwent endoscopic ultrasound examination, and the size of the lesion was measured based on the excised specimens.

Two patients with smaller lesions underwent Cap-EFTR, while the remaining patients adhered to the EFTR protocol [15], which included marking, submucosal injection, circumferential incision, and lesion dissection.

Main equipment included gastroscopes (Q260J/PCF-H290L/I Olympus, Tokyo, Japan), Hook knife (KD-620LR; Olympus), disposable mucosal incision knives (MK T-2–195-N, Micro-Tech), IT knife (Olympus), conventional TTSCs (R-C/D series; Micro-Tech Co, Ltd, Nanjing, China), DATs (STA00002, Micro-Tech Co, Ltd, Nanjing, China), injection needles (22G, Micro-Tech) and so on.

### DAT closure procedure

- Hold the tissue at the edge of the wound with one arm of the DATs.
- Pull the clamped tissue toward the opposite edge and clamp the opposite tissue with the other arm of DAT.
- Release DAT to close the wound after both edges are clamped.
- The DAT is utilized to reduce the size of the wound by transforming a large wound into a smaller one, after which the remaining small wound is sutured using TTSCs.

Notably, the DAT can effectively clamp mucosal, muscular, or full-thickness tissue at the wound edge, following essentially the same procedural steps across different tissue layers.

### Data collection

The data collected included patient demographics, lesion location, operation duration, lesion size, wound closure time, average operation time for each DAT, intraoperative and postoperative complications, the number of DAT/TTSC utilized, pathology, postoperative hospital stay, total hospital stay, and antibiotic duration. Intraoperative and postoperative complications primarily included bleeding and perforation. Complications specific to the DAT procedure mainly involved mucosal tears, bleeding, and perforation, with a 3-month follow-up conducted for any delayed perforation or bleeding. Instances of DAT/TTSC detachment were documented during endoscopic follow-up. The total operation time was defined as the duration from submucosal injection to the release of the last clip. Suture time referred to the period from the placement of the first clip to the release of the last clip. Perforation duration time was defined as the interval from intraoperative perforation to completion of suturing. The suturing process commenced when the first clip was inserted into the stomach, and the operation was deemed complete upon successful clip release. Lesion size was measured from the resected specimen, and the wound size was estimated to be equivalent to the lesion size.

### Statistical analysis

Descriptive statistical analysis was conducted to characterize the changes from the baseline. Quantitative data are presented as median (interquartile range), while categorical data are expressed as frequency (percentage). All statistical analyses were performed using IBM SPSS Statistics for Windows, version 27.0 (IBM Corp., Armonk, NY, USA).

## Results

**Figure 2:**
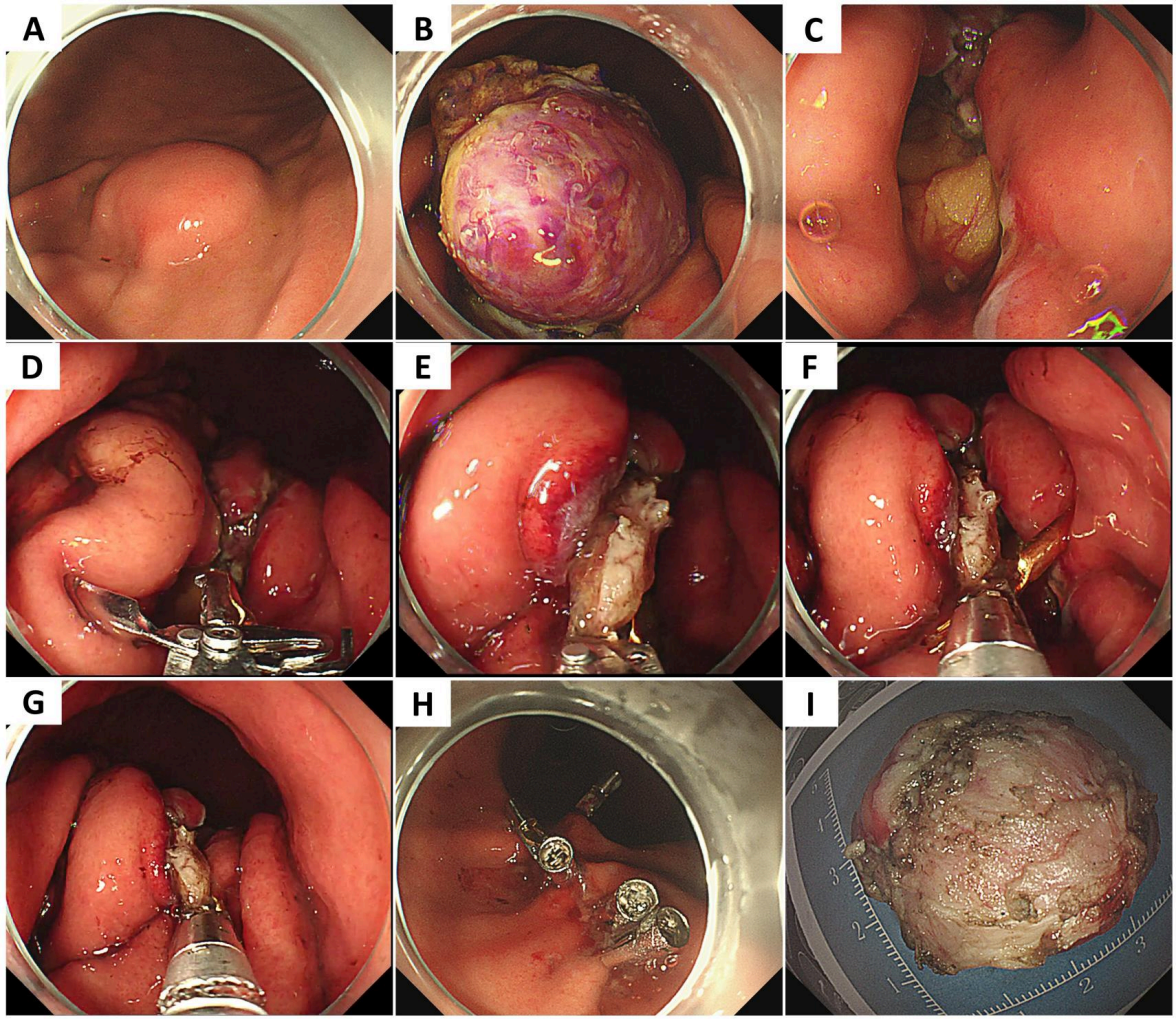
Closure of a gastric perforating wound using DAT combined with TTSCs. The lesion was located in the greater curve of gastric body with a size of 3.2 × 2.5 cm. (A) Endoscopic view of gastric submucosal tumors originated from muscularis propria. (B) The tumor was fully exposed with an intraoperative perforation. (C) The peritoneum can be observed through the perforated wound. (D) The appearance of DAT can be observed. (E) Hold the tissue at the edge of the wound with one arm of the DAT. (F) Pull the clamped tissue toward the opposite edge and clamp the opposite tissue with the other arm of DAT. (G) Pull up the DAT to check if both edges are clamped and then release the DAT. (H) The gastric wound was closed with DAT and TTSCs successfully. (I) Postoperative specimen photographs. DAT, dual action tissue closure device; TTSC, through-the-scope clip.

**Figure 3:**
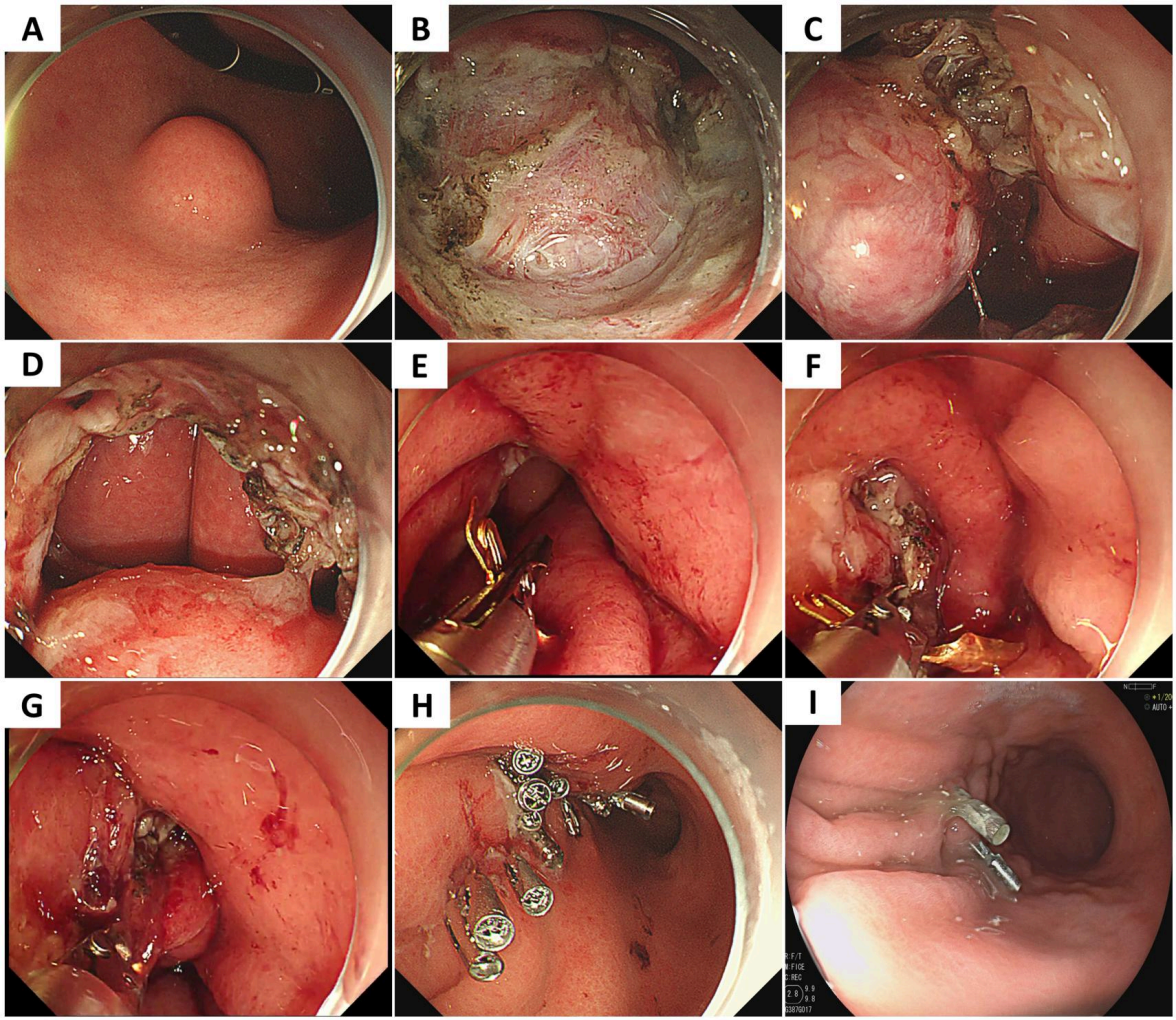
Closure of a gastric perforating wound using DAT combined with TTSCs. The lesion was located in the anterior wall of the gastric body with a size of 5.0 × 3.5 cm. (A) Endoscopic view of gastric submucosal tumors originated from muscularis propria. (B) Incision into muscularis propria around the lesion. (C) Incision into serosal layer around the lesion was performed to create active perforation. (D) The lesion has been completely removed and the wound has perforated. (E) The appearance of DAT can be observed. (F) Hold the tissue at the edge of the wound with one arm of the DAT and pull the clamped tissue toward the opposite edge and clamp the opposite tissue with the other arm of DAT. (G) Pull up the DAT to check if both edges are clamped and then release the DAT. (H) The gastric wound was closed with 4 DATs and TTSCs successfully. (I) The patient was followed up via endoscopy 5 months later. All of the DATs had detached, leaving only two TTSCs remaining. DAT, dual action tissue closure device; TTSC, through-the-scope clip.

In this study, all EFTR perforation wounds measured > 1 cm, with 48.2% exceeding 2 cm, and were successfully closed using DAT and TTSC. As presented in Tables 1 and 2, this study included a total of 29 cases. Among these cases, 17 lesions were located in the gastric fundus. All resection wounds were classified as perforation-type wounds, which were successfully sutured using DATs and TTSCs, resulting in a 100% closure rate. The median postoperative hospital stay was 4.0 days (interquartile range: 2.5–5.0 days), with dietary intake resumed after a median of 3.0 days (interquartile range: 2–3.5 days) and antibiotic treatment administered for a median of 4 days (interquartile range: 3–5 days). The long diameter of the wounds measured a median of 20 mm (interquartile range: 15–30 mm), while the short diameter was 15 mm (interquartile range: 10–25 mm). The median time required for wound closure was 19 minutes (interquartile range: 12–27 minutes), and the total operation time averaged 55.0 minutes (interquartile range: 33.5–70.0 minutes). The perforation duration time averaged 31.0 minutes (interquartile range: 18.5–47.5 minutes). At the 3-month postoperative follow-up conducted via telephone, none of the patients reported delayed bleeding, perforation, or infection. Seven patients underwent endoscopic follow-up at a median of 142 days (interquartile range: 75–201 days), revealing that all wounds healed well. Among these seven patients, a total of 17 DATs were utilized; however, two DATs failed to detach in two patients, resulting in an overall detachment rate of 88.2%, while 21 of 53 TTSC remained (detachment rate: 39.6%). Figures 2 and 3 demonstrated the suturing of larger gastric perforations, indicating the DAT’s effectiveness for large perforations. Videos S1 and S2 (in Supplementary material) demonstrated two cases of gastric perforation after EFTR that were closed using the DAT and TTSCs, with good suturing outcomes. Pathological examination of the resected specimens revealed that 20 cases (69.0%) were stromal tumors, classified as follows: 13 cases of very low risk, 3 cases of low risk, 1 case of medium risk, and 3 cases of high risk. Patients with medium and high risks were prescribed oral imatinib and are currently under regular follow-up.

**Table 1:** Characteristics of patients, lesions, and procedures in this study.^a^

Characteristics	Values
Age, years	57.0 (46.0–64.5)
Female	11 (37.9)
Body mass index, kg/m^2^	22.9 (21.5–24.0)
Lesion location	
Fundus	17 (58.6)
Anterior wall	10 (58.8)
Posterior wall	7 (41.2)
Body	11 (37.9)
Antrum	1 (3.5)
Lesion size	
Long diameter, mm	20 (15–30)
Long diameter	
< 20 mm	11 (37.9)
20–30 mm	10 (34.5)
≥ 30 mm	8 (27.6)
Short diameter, mm	15 (10–25)
Short diameter	
< 20 mm	15 (51.7)
20–30 mm	11 (37.9)
≥ 30 mm	3 (10.3)
Lesion histopathology	
Gist	20 (69.0)
Very low-risk	13 (65.0)
Low-risk	3 (15.0)
Medium-risk	1 (5.0)
High-risk	3 (15.0)
Leiomyoma	1 (3.4)
Neurinoma	5 (17.2)
Non-neoplastic	3 (10.3)
Type of endoscopic resection	
Cap-assisted full-thickness resection	2 (6.9)
Endoscopic full-thickness resection	27 (93.1)

a Values are shown as median (interquartile range) or *n* (%).

**Table 2:** Clinical outcomes of the 29 patients in this study.^a^

Characteristics	Values
Total procedural time, mins	55.0 (33.5–70.0)
Clip closure	
Complete clip closure	29 (100)
Clip closure time, mins	19 (12–27)
Average operation time for each DAT, mins	3.0 (1.6–6.5)
Perforation duration time, mins	31.0 (18.5–47.5)
Number of clips used for complete clip closure	
DAT	1 (1–3)
TTSC	10 (7–12)
Postoperative management	
Abdominal paracentesis	2 (6.9)
Gastric tube intubation	15 (51.7)
Postoperative hospital stay, days	4.0 (2.5–5.0)
Analgesic medication use	7 (24.1)
Total hospital stay, days	8 (7–9)
The time for resuming diet after surgery, days	3.0 (2.0–3.5)
The duration of antibiotic use, days	4 (3–5)
Adverse events	
Delayed bleeding	0 (0)
Delayed perforation	0 (0)
Serious infection	0 (0)
Endoscopic follow-up period, days	142 (75–201)
Number of clips left	
DAT	2/17 (11.8)
TTSC	21/53 (39.6)

a Values are shown as median (interquartile range) or *n* (%). DAT, dual action tissue closure device; TTSC, through-the-scope clip.

In practice, two patients underwent abdominal paracentesis during surgery for abdominal decompression. A total of 7 doses of tramadol were given postoperatively to 5 patients for postoperative pain relief and 15 patients had nasogastric tubes placed for postoperative decompression.

## Discussions

In this study, all EFTR perforation wounds were successfully closed using DAT and TTSC. Although the perforation lasted 31 minutes (range: 18.5–47.5 minutes), the patient had an uneventful postoperative course without any complications. This suggests that DAT represents a safe and effective method for suturing perforations caused by ESD.

Zhou *et al.* [15] were the first to report the use of TTSC for closing EFTR defects in 26 cases, where the average lesion size was 2.8 ± 1.3 cm and the average operation time was 105 minutes (range: 60–145 minutes). When the defect was slightly larger than the clip width, air aspiration was employed to reduce the size before clamping. For larger defects, the omental-patch method was utilized [16]. However, aspiration-based closure and the omental-patch method are less precise and more challenging than DAT. But the article does not specify the wound suture time. Another study focused on smaller lesions (1.59 ± 1.01 cm), reporting an average operation time of 59.7 minutes (range: 30–270 minutes), with wounds sutured using TTSC [17]. In contrast, our study included lesions measuring 20 mm (range: 15–30 mm), the size of the lesion is intermediate relative to the findings of the two aforementioned studies, with a total operation time of 55 minutes (range: 33.5–70 minutes), which has a shorter operation time.

The endoscopic purse-string suturing is recognized as both safe and effective [18], albeit technically demanding. A study involving 68 EFTR cases reported lesions measuring 2.60 ± 0.5 cm (range: 2–3.0 cm) and indicated an average closure time of 13 minutes (range: 9–21 minutes) and a total operation time of 41 minutes (range: 23–118 minutes), with one case of delayed bleeding [19]. In contrast, another study [20] involving 28 lesions averaging 1.9 ± 1.1 cm reported an operation time of 55.7 ± 15.4 minutes, but the closure time was not mentioned. Both studies achieved a 100% closure rate. In our analysis of 29 EFTR cases, 58.6% of lesions were located in the gastric fundus, achieving a 100% closure rate with a total operation time of 55.0 minutes (range: 33.5–70.0 minutes) and no complications. Regarding the above studies, there seems to be no significant advantage in the total operation time. This is influenced by multiple factors such as operating experience, surgical difficulty, and lesion location. In this study, the time for wound closure using DAT combined with TTSC was 19 (12–27) minutes. Additionally, the use of endoscopic purse-string suturing typically requires retraction of the tissue for installation. A learning curve is also present, particularly for less experienced surgeons. Furthermore, when utilizing TTSC to secure the nylon rope to the wound’s edge, any tilting of the TTSC towards the wound can impede the effective tightening of the nylon rope, thereby significantly impacting operational efficiency. For EFTR perforated wounds, prompt closure is critically important. DAT can directly pass through the endoscopic channel without the need to retract the endoscope, which demonstrates significant potential advantages. In this study, all perforation wounds were larger than 1 cm, with 48.2% exceeding 2 cm in size. The median operation time for DAT alone was only 3 minutes, with each wound requiring 1 (1–3) DATs. After converting larger wounds into smaller ones, TTSC was employed to close the remaining wounds. Furthermore, various suture devices have been reported, including the mantis clamp, overstitch, and X-tack. These devices serve complementary roles depending on the characteristics of the wound. For closing perforation wounds, X-tack can effectively close perforation wounds via the endoscopic working channel and is particularly suitable for large wounds, especially in cases where the tissue at the wound edges is swollen and scarred [21]. The mantis clamp, a newly reported endoscopic clip inspired by the forelegs of the mantis, can perform “grabbing-dragging” operations on gastric mucosal defects to effectively close the wound. It presents a potential alternative closure device for large perforation wounds [22].

Recently, a pilot study comparing the use of DAT combined with TTSC vs the use of TTSC alone for suturing EFTR wounds included 19 cases [23]. The study demonstrated that DAT could enhance the suture efficiency. However, the lesions included in this study were relatively small, with the DAT group having an average size of ∼1.3 ± 0.3 cm in size. Based on our experience, DAT appears to offer a greater advantage in closing larger perforation wounds. Nevertheless, the size of the lesions in this study ranged from 20 to 30 mm, which undoubtedly further improves the application research of DAT.

This study has several limitations. First, it is a single-center, retrospective, single-arm study, which reflects only our own experience and provides a preliminary evaluation of the reliability and safety of DAT usage. Second, the study measured the size of the excised tumor specimens as a proxy for the size of the perforation wound, which may not accurately represent the actual wound size. After EFTR surgery, gas enters the abdominal cavity, compressing the wound to some extent, making precise measurement challenging. Additionally, the prompt closure of the wound following perforation is a primary consideration. Consequently, these limitations are difficult to completely mitigate in this study. Third, this research did not include a direct comparison with existing technologies, such as nylon suture purses, over-the-scope clips and X-tack, each of which has its own advantages and disadvantages, and these instruments can be considered complementary. Fourth, similar to traditional endoscopic clips, the TTSC and DAT also detach and exit the body as the wound heals. In this study, only 24% of patients underwent endoscopic follow-up, which is a relatively small proportion. Notably, 88.2% of the DATs detached within a follow-up period of 142 days (range: 75–201 days).

Finally, there may be some weakness in the cases included. In this study, 58.8% lesions were located in the gastric fundus. This is primarily because during gastric ESD and EFTR procedures, lesions in the gastric fundus have a higher probability of perforation. A retrospective study showed that the perforation risk for gastric fundus stromal tumors was 4.9 times higher than in other locations [24]. A multicenter prospective cohort study in Japan revealed that perforations most frequently occur in the upper third of the stomach, with a perforation rate of 7.9%, which is higher than the average rate (2.0%) [25]. Meanwhile, endoscopic repair of gastric fundus perforation is particularly challenging, primarily attributed to extreme retroflexion, thin muscular layer and limited working space [26].

## Conclusions

This study preliminarily demonstrates that the combination of DAT and endoscopic clips for closing perforation wounds resulting from EFTR is both reliable and safe. This approach represents potential alternative closure method for wounds exceeding 10 mm. Further prospective randomized controlled trials are warranted to validate these findings.

## Supplementary material

Supplementary material is available at *Gastroenterology Report* online.

## Authors’ contributions

Q.Z. designed the study; Q.Z., J. Cai and L.M completed all the endoscopic submucosal dissection surgeries; L.M and Y.M drafted the manuscript and collected the data; S.L. collected the data; Y.X. completed the data analysis; Z.W., B.Z., Z.G., J. Chen, D.Z., P. L., T.X., and S.W. participated in the surgery.

## Supplementary Material

goag070_Supplementary_Data
